# Development and testing of a game-based digital intervention for working memory training in autism spectrum disorder

**DOI:** 10.1038/s41598-021-93258-w

**Published:** 2021-07-05

**Authors:** Surbhit Wagle, Arka Ghosh, P. Karthic, Akriti Ghosh, Tarana Pervaiz, Rashmi Kapoor, Koumudi Patil, Nitin Gupta

**Affiliations:** 1 Department of Biological Sciences and Bioengineering, Indian Institute of Technology Kanpur, Kanpur, Uttar Pradesh 208016 India; 2 Amrita School for Special Children and Rehabilitation Center, Kanpur, Uttar Pradesh 208005 India; 3 Regency Hospital Limited, Kanpur, Uttar Pradesh 208005 India; 4 Design Program and Department of Humanities and Social Sciences, Indian Institute of Technology Kanpur, Kanpur, Uttar Pradesh 208016 India; 5 Cognitive Science Program, Indian Institute of Technology Kanpur, Kanpur, Uttar Pradesh 208016 India; 6 Mehta Family Center for Engineering in Medicine, Indian Institute of Technology Kanpur, Kanpur, Uttar Pradesh 208016 India

**Keywords:** Human behaviour, Rehabilitation, Rehabilitation, Autism spectrum disorders

## Abstract

Autism spectrum disorder (ASD) is prevalent globally, yet it lacks cost-effective treatment approaches. Deficits in executive functions occur frequently in autism spectrum disorder and present a target for intervention. Here we report the design and development of five smartphone-based games for training working memory in children with ASD. These open-source games, available free of cost to the community, were designed to match the behavioral preferences and sensorimotor abilities of children with ASD. We then conducted a preliminary trial to test the effectiveness of a month-long intervention using these games. Although we did not see a significant change in the working memory of all children with a month-long training, children who performed better on the games also showed more improvement in their working memory, suggesting that a longer intervention with the games might be useful in improving working memory. Using a Hindi translation of the autism treatment evaluation checklist, we also tested the collateral gains of the training in reducing autistic symptoms. We found no significant change in the autistic symptoms after the intervention. Further, there was no correlation between the change in the working memory and the change in the autistic symptoms.

## Introduction

Autism Spectrum disorder (ASD) is a neurodevelopmental condition characterized by problems in communication, social interactions, idiosyncrasy, and repetitiveness in behaviors^1^. According to WHO estimations, 1 in every 160 children worldwide is on the ASD^2^. The country-specific prevalence rates are 1.70% in the USA^3^, 2.64% in South Korea^4^, and 0.23% in India^5^; lower rates in developing countries like India may partly be due to lack of awareness and diagnoses. Current treatments for ASD include early interventions like Applied Behavioral Analysis^6,7^. These treatments are cost- and time-intensive^8,9^, and hence often limited in their access, particularly in developing countries. There is a pressing need for developing and testing new cost-effective treatment approaches for ASD.

Executive functions (EFs)—cognitive processes such as working memory (WM), attention, cognitive flexibility, planning, set shifting—are essential for the day-to-day functioning of people. Individuals with neurodevelopmental conditions are likely to have some deficits in EFs, although the deficits may differ across the affected individuals^10^.

Baddeley and Hitch^11^ described WM as the processes by which information is temporarily stored and processed, enabling the execution of complex tasks such as learning, language comprehension, and reasoning. According to this model, WM consists of three components, the visuospatial sketchpad, which is responsible for maintaining image-related and spatial information; the phonological loop, which is responsible for storing speech-based information; and the central executive, which acts as a controller for maintenance of task-relevant focus and processing abilities^12^. Some studies found that individuals with ASD show impairments in both verbal and visuospatial WM^13^, while others found that visuospatial WM is more severely impaired^14–16^. WM interacts with other executive processes that are necessary for everyday activities such as cognitive flexibility and planning^12^. In some individuals with ASD, WM deficits could result in several problems related to cognitive flexibility, sustained attention, abstract thinking and behavior regulation^17–19^. WM is also crucial for various academia-related skills such as problem solving^20^, arithmetic^21^, and reading^22^. In a recent study, Schuh et al.^23^ reported WM deficits in high-functioning autistic (HFA) children compared to their age, IQ and language matched typically developing peers; in the HFA group, the WM ability predicted the autistic symptom severity^23^. WM impairments are also related to learning disabilities, other EF deficits^24^, and restricted and repetitive behavior^25–27^. One study found that high functioning ASD individuals made more between-search errors and employed a less strategic search compared to their typically developing (TD) peers^27^. Several studies have reported that in ASD individuals verbal WM impairments increase with an increase in WM load and are absent when the load is minimum^16,28^. Similarly, for the visual-spatial WM, studies have reported significantly poorer performance of the ASD groups compared to the TD control group in spatial working memory tasks^29^. However, other studies have reported comparable performance in the two groups^30–34^. These inconsistencies might be due to the differences in the severity of ASD in the participants^35^ and the type of WM (verbal or visuospatial) examined^24^. A recent meta-review concluded that, on average, ASD individuals have deficits in verbal and visuospatial working memory and these deficits are present in individuals in a broad age range^15^.

Effects of EF training for clinical and TD populations have been investigated previously^36–38^. Several studies have looked into the benefits of using computerized WM training for ADHD, learning disability, and TD populations^39,40^. These studies found a significant near-transfer effect to WM, the trained EF, although the collateral gains to other EFs were less clear^41^. Improvements in cognitive abilities in young children with ASD can concurrently show improvement in their social skills^42^. This raises the possibility that WM training may reduce ASD symptoms, since studies have shown a link between WM and the repetitive and restricted behaviors in ASD^25–27^. Baltruschat et al. were able to show improvements in WM task performance of autistic individuals with the help of positive reinforcement^38,43^. They used items that were highly preferred by these individuals as the positive reinforcement. Also, the high performance was maintained at follow-up, when the reinforcement was removed^38,43^. This suggests an opportunity to train WM using incentives that are preferable to ASD population.

Despite the evidence of WM deficits in ASD population and of positive results of WM training in similar conditions, very few studies have explored WM training as a potential intervention for ASD. In a recent study, effects of computerized working memory training or cognitive flexibility training, over 6 weeks, were compared against a mock training in ASD children^44^. At post-training, both the experimental groups showed near-transfer improvements in their respective trained EFs. The working memory training showed marginal improvement in attention at post-training^44^. The study, however, suffered from a relatively high number of dropouts.

We hypothesized that training visuospatial working memory in autistic children could ameliorate the severity of symptoms in children affected with ASD. The objective of the current study was to develop mobile-based games that trained visuospatial WM and conduct a month-long trial as a preliminary assessment of the feasibility and efficacy of our games in improving the WM and autistic symptoms in ASD children.

We developed a suite of mobile-based games to train visuospatial WM in autistic children. Our aim was to develop simple and interesting games that are able to engage children with ASD and are freely available to all children. Although the games were based on the English language, only the simplest words were used and the use of language was kept to a minimum so that the games can be used by non-native English-speakers globally. To incentivize gameplay, our games were designed to align with behavioral preferences of some ASD children, and were improved using iterative user testing. Some of our games are based on the idea of color and shape matching. During our initial prototype testing we observed that the children were able to understand these ideas easily, as their school-based activities frequently involve (without any WM component) matching of shapes on paper and coloring an empty object with the same color as that of another object. In the games, objects of specific colors or at specific spatial locations are shown briefly and then hidden, thus requiring visuospatial memory to solve the task. Our games also included the concepts of shape-integration and remembering spatial sequences. We conducted a preliminary study to test the effectiveness of a short-term (30 min per day for 1 month) intervention using our games in improving WM and reducing autism severity.

## Methods

### Game development process

We sought to develop a suite of five games for providing working memory training. The games were designed with sensorimotor and behavioral components to incentivize and simplify gameplay for children with ASD. It has been observed that autistic children prefer green and brown and show slight aversion towards the yellow color^45^. Accordingly, we reduced the use of yellow in the games and instead made more game elements using the preferred colors. We kept navigation in the games simple to keep the gameplay accessible to most children: for instance, the games mostly required tapping of objects instead of dragging. Considering the short visual attention spans of autistic children, the games were designed to have single windows with very few elements on the screen to minimize distractions. An earlier study showed improvement in task engagement when the directives were sung instead of spoken^46^. Therefore, we used song-like intonations in complimentary messages such as “well done” and “you are awesome”, which are provided when the child successfully completes different steps in the games. Lining up toys is one of the common repetitive behaviors observed in autistic children^47^. We exploited this preference to motivate the children by designing gameplays where the game objects get arranged in a line as the player progresses through the game. We further optimized our game development process based on the inputs from parents and caretakers of autistic children, and based on a survey of existing games (and their reviews) on the Google Play and the iTunes store. We iteratively refined the initial versions of the games by user testing with the children and by taking feedback from the therapists at the Amrita School for Special Children, Kanpur, India.

The games were programmed to run on smartphones or tablet devices with the Android Operating System. We targeted Android, as it is the most widely used mobile operating system globally. The games were developed using Unity 2018.3.5f1 game engine. The games are made freely available on the Play Store at this link: https://play.google.com/store/apps/details?id=org.TreadWill.WorkingMemoryGames&hl=en. The source code of the games is also made freely available at this link: https://github.com/neuralsystems/working-memory-training-games.

### Participants

All participants were recruited from Amrita School for Special Children (AmritaSSC), Kanpur, India. 19 parents responded to our request for participation in this study. After an initial screening to exclude children with serious behavioral problems who could not use tablet devices, 14 children between the age of 6–13 were included in the trial as participants (see Table 1). Thirteen out of the fourteen children were diagnosed with ASD by a physician or a therapist at the center using DSM-5 criteria^1^, while one had a diagnosis of Down’s syndrome with some symptoms of ASD⁠. None of the participants received formal education in a regular school. Eleven participants were students in the special-needs school run by AmritaSSC. The remaining three participants visited the center for behavioral therapy and one-to-one interactions with the teachers of the school. The students visited the center for an average of 5 days/week. The non-student participants visited the center in varying frequencies: of the three non-student participants, one visited 5 days/week, and two visited 2 days/week. Five participants were diagnosed with other comorbidities (see Table 1). All of the participating children received the same intervention.

**Table 1:** Characteristics of participants.

Characteristic	Participants (n = 14)
Mean age (years)	10.24 (1.92)
Gender (male: female)	11:3
ASD diagnosis	13
Down Syndrome	1
Student (S): Non-student (NS)	11: 3
**Comorbidities (n = 5)**	
Intellectual disability	2
Mental retardation	1
Developmental delays	2
**Education level of students in AmritaSSC curriculum (n = 11)**	
Level 1	1
Level 2	2
Level 3	3
Level 4	5
**Training intensity (n = 14)**	
5 sessions/week (Student: Non-student)	12 (11:1)
2 sessions/week (Student: Non-student)	2 (0:2)

### Outcome measures

To determine the effectiveness of our games, we evaluated the change in the working memory and the collateral gains related to the autistic symptoms after training. These two effects were examined using the measures described below.

#### Working memory

To record changes in visuospatial working memory, the standard in-person Corsi-block tapping task^48^ was administered. The task apparatus consisted of nine black-colored blocks randomly placed on a stage. In a single trial of the task, an examiner tapped a sequence on the blocks and the participant was instructed to repeat the tapped sequence as soon as the examiner finished tapping. The participant was instructed to refrain from tapping until the examiner finished tapping. The task started with an initial sequence length of two blocks and had a maximum sequence length limit of nine blocks. We tried two trials for each sequence length and used a different sequence in each trial. If the participant failed to produce at least one of the two sequences of same length correctly, the task was ended. If the participant correctly reproduced the sequences on the two trials, the sequence length was increased by one. Two dependent parameters in the task were noted. The first parameter is the length of the longest correctly produced sequence, also known as the Block span, and is commonly used for measuring performance on this task. The other parameter is the product of the Block span and the total number of correctly produced sequences, also known as the Total score. The latter is considered more reliable and sensitive to minor changes in the task performance^48^. We have used both parameters as the outcome measures of the task.

#### Collateral gains related to ASD symptoms

The Autism Treatment Evaluation Checklist (ATEC)^49^ is a popular tool to measure the effectiveness of an intervention for patients on the autism spectrum. The ATEC form consists of 77 items that cover four major categories of difficulties observed on the autism spectrum. These categories: (1) Speech/Language/Communication (14 items); (2) Sociability (20 items); (3) Sensory/Cognitive Awareness (18 items); (4) Health/Physical/Behavior (25 items). The first three categories are rated on a 0–2 scale and the fourth category is rated on a 0–3 scale. Parents provided a rating for each item, which resulted in a total score between 0 and 179. This total score was used as the outcome measure of the ATEC. Since most of the parents were more comfortable with the Hindi language, we provided a Hindi translation of the ATEC for assessment (available at https://github.com/neuralsystems/ATEC_Hindi).

To assess the outcome of the working memory training, the above-mentioned measures were taken before and after the intervention for all the participants. Both assessments were taken within 1 week of the start and the completion of the intervention.

### Intervention

During the training session, the participant was presented with a headphone-plugged tablet with the game suite installed in it. All training sessions were monitored by an experimenter. In a session, the child had the liberty to play any of the games in the suite. The child was allowed to switch between the games as many times as he or she wanted, although the experimenter monitoring the session verbally encouraged the child to avoid switching too many times and play a game long enough to understand the gameplay and learn from it. Each training session lasted for at most 30 min. During the sessions, the experimenters noted the response of the participants to different game features.

### Procedure

The study was approved by the Institute Ethics Committee of the Indian Institute of Technology Kanpur, and was performed in accordance with relevant guidelines. Informed consent for participation in the study was obtained from the parents of the children. Subsequently, the participant was administered the pre-treatment Corsi task, by either an experimenter or the participant’s favorite teacher at the center. For each participant, the same person administered the task pre-intervention and post-intervention so that any difference due to familiarity with the task administrators can be avoided. The teachers received a tutorial about the task procedure before they administered the task to any of the participants. For all the participants, their parents filled the pre- and the post-training ATEC form. For 13 out 14 children, the pre- and the post-treatment ATEC form was filled by the same parent. For one child, the mother filled the post-intervention form due to long-term inaccessibility of the father who had filled the pre-intervention form. Participants who were students in the school were provided the training during their school hours, depending on their availability from other school activities. The non-student participants were provided the training during their visit to the center for the therapy session. The methods are performed in accordance with approved guidelines and regulations. As the study was conducted as a preliminary trial, it was not registered prospectively; it was retrospectively registered at clinicaltrials.gov (identifier number NCT04308915, date of registration: 16/03/2020).

### Data analysis

Given the small sample size available for our study, we did not assume the data to be normally distributed and used nonparametric tests to assess the statistical significance of the results. The analyses were performed in MATLAB. We calculated the change in Block Span as the difference in its values at the post-intervention and the pre-intervention time points. Similarly, the change in the total scores of Corsi task was calculated. Non-parametric Wilcoxon signed-rank tests were used to determine if the median change among the 14 participants is significantly different from zero (results from the parametric *t*-test and Cohen’s *d* for effect size are provided in Supplementary Table S1). To determine the significance of the change in autistic symptoms, Wilcoxon signed-rank test was applied to the changes in the ATEC scores. Spearman rho values were used to assess the correlations between the changes in different outcome measures. For determining statistical significance, we set the *P* value threshold to 0.05.

## Results

### Development of games for working memory training

The design process described in the Methods resulted in five games for working memory training in ASD children.

#### Basket game

**Figure 1:**
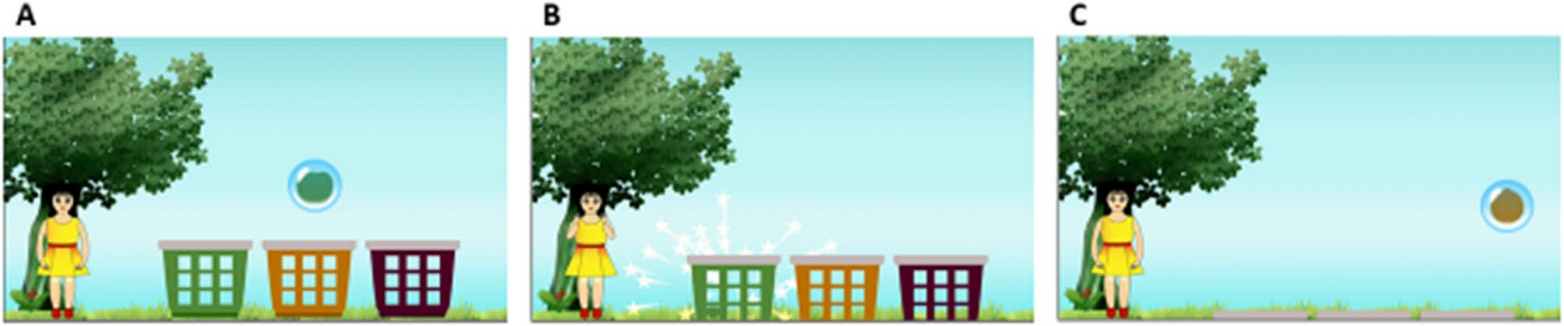
Basket game gameplay. (**A**) A colored fruit is trapped inside a bubble, which moves in a zigzag path above the differently colored baskets. (**B**) The fruit falls in one of the baskets when the player bursts the bubble by tapping it. If the color of the fruit matches the color of the basket, a visual reinforcement is provided: the girl on the left claps and laughs, and a particle scattering effect is displayed. (**C**) As the game progresses, the baskets move down (and out of the screen) gradually such that eventually the colored parts of the baskets are completely hidden.

The objective in the game is to drop colored fruits into baskets of matching color. The player must tap on a moving bubble, containing a colored fruit, only when the bubble is above the basket of the same color (Fig. 1). An audio-visual animation, which many children with ASD find rewarding, is presented if the fruit falls in the correct basket. After a few correct plays, the colored part of all the baskets is hidden, thus requiring the player to remember the colors of the baskets at different positions. As the user is able to perform well, the difficulty in the levels is increased by increasing the number of baskets and the number of fruits per basket. We also added an introductory level to the game, in which the user learns the idea of collecting fruit into the basket. The introductory level is divided into three stages. In the first stage, we show a single basket and one static fruit in a bubble. Tapping on the bubble bursts it and the fruit is collected in the basket. After several correct trials, for the second stage, we add movement to the fruit such that it starts moving in a zigzag path, starting from the bottom left corner of the screen. For a few trials, the fruit moves until it reaches above the basket and stops there. In the last few trials of the introductory level (stage three), the fruit does not stop above the baskets but continues to move. Thus, we gradually teach the kid the idea of collecting moving fruits into the basket. The participant had to complete all the three introductory stages in one go to clear it. In the introductory level, we removed other all the objects from the screen to keep the attention of the child on learning the objective of the game.

#### Train game

**Figure 2:**
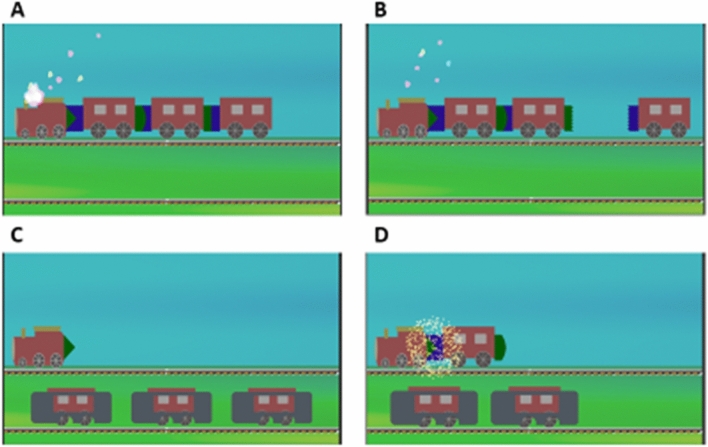
Train game gameplay. (**A**) A train moves into the scene with smoke animation and sound. (**B**) Each wagon detaches from the train and moves to a random position on the rail track below. (**C**) After some delay, the shapes at the ends of the wagons are hidden by a cover. The player is then prompted to select the wagon that has the shape complementary to the last wagon on the train. (**D**) Selection of the correct wagon results in elongation of the train and is rewarded by an audio-visual feedback.

The objective in the game is to attach train wagons, each with specific shapes on its two ends, such that any two adjacent wagons have complementary shapes fitting snugly into each other (Fig. 2). Correct play results in aligning of wagons and elongation of the train, which act as an incentive for the autistic children, who often have an inclination to arrange toys in lines. In the initial levels of the game, the shapes of the wagons are kept visible so that the player can understand the game play. As the player progresses to harder levels, the shapes of the wagons are hidden after being shown for a short duration, thus requiring the child to remember the shapes at different positions. Based on the feedback received in initial testing, we have also added some introductory levels in the game to teach the idea of matching complementary shapes (without wagons or other stimuli on screen) so that the children can slowly acquire the minimum skills required to play the game. The introductory level includes five stages in which we presented different 2-D shapes. After a short delay, a piece of the original shape detaches and moves to the right side of the screen. Two other pieces are also presented on the right side, along with the detached piece, as choices to fill the gap in the original shape. If the user selects the correct piece, it moves to the original shape, and a “click” sound is played to indicate that the piece fits perfectly into the shape. As the introductory stages progress the complexity of the missing piece is increased. The participant had to finish all the five introductory stages in one go to clear them.

#### Piano game

**Figure 3:**
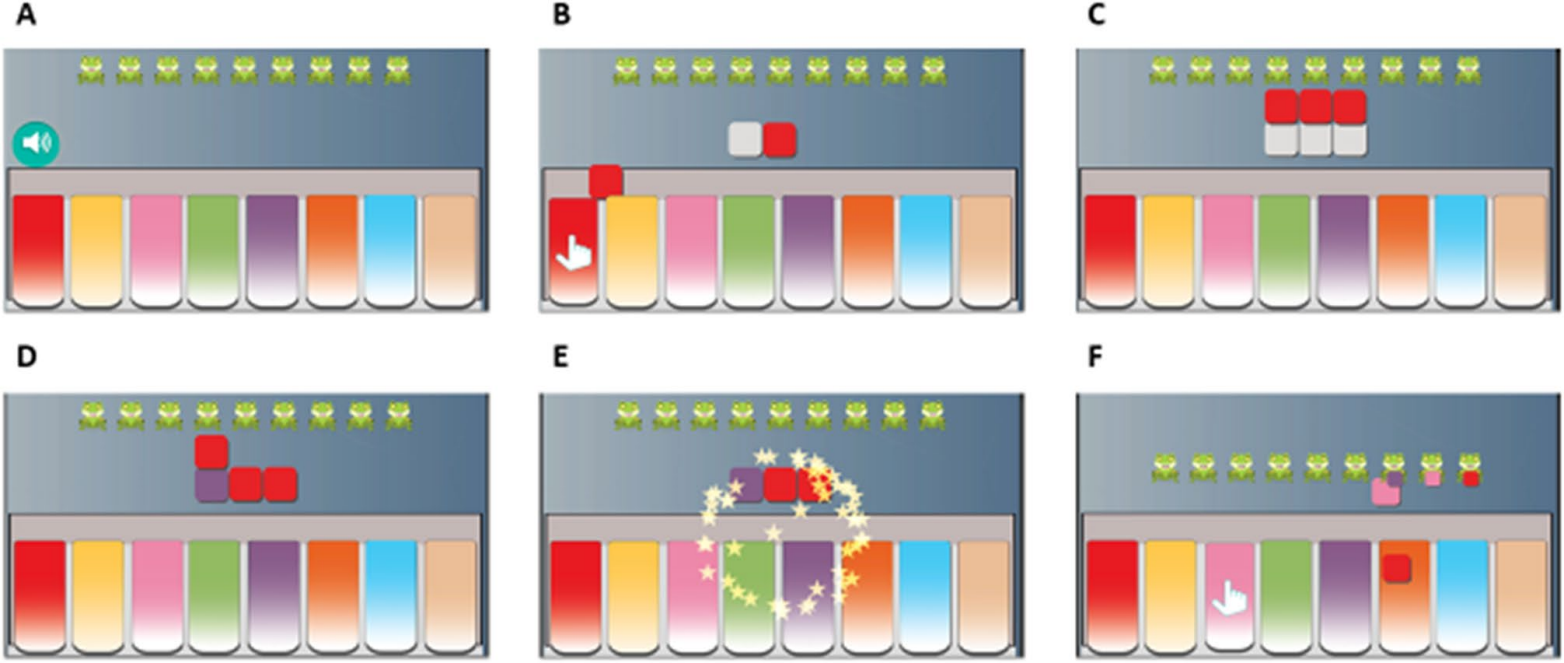
Piano Game gameplay. (**A**) The player clicks on the speaker icon to make the computer play a target sequence. (**B**) When the target sequence is played, tiles of the same colors as the played keys are generated and aligned above the keyboard. In addition, a visual cue (hand) indicates the pressed keys. After a delay, the colors of the tiles are hidden (color turns to white). Now, the player is prompted to repeat the target sequence using the keyboard. (**C**) As the player presses the keys, tiles corresponding to the pressed keys are arranged on top of the tiles of the target sequence. (**D**) When the player finishes repeating the sequence, the colors of the target tiles are uncovered, and the matches between the target and the player’s tiles are displayed. (**E**) An audio-visual reinforcement is provided if there is a complete match, and the play starts again with subsequent notes from the same song. The correctly played tiles also get added to a sequence of frogs at the top part of the screen. (**F**) At the end of a level, when the user has completed multiple sequences of a given length, the full song is played with animation. The user then progresses to the next level with longer sequences.

The objective in the game is to hear and then replay a short sequence of musical notes on a virtual piano on the screen (Fig. 3). This game capitalizes on the penchant of autistic children towards music. The note sequences are taken from popular songs such as “Baa Baa Black Sheep” and “Humpty Dumpty”. We have used visual animations to make it easy to see the pressed keys. A pleasant audio-visual animation is displayed when the player correctly repeats the sequence; the correctly played keys are also added to an elongating line on top to further motivate autistic players. We do not provide any feedback for incorrect play because we observed during the testing phase that, for some children, any kind of audio or visual feedback (even if given after mistakes) was enjoyable and could encourage them to repeat mistakes during the game. When the child is able to perform well, we increase the difficulty of the game by increasing the length of the sequence and the number of trials per sequence length.

#### Face game

**Figure 4:**
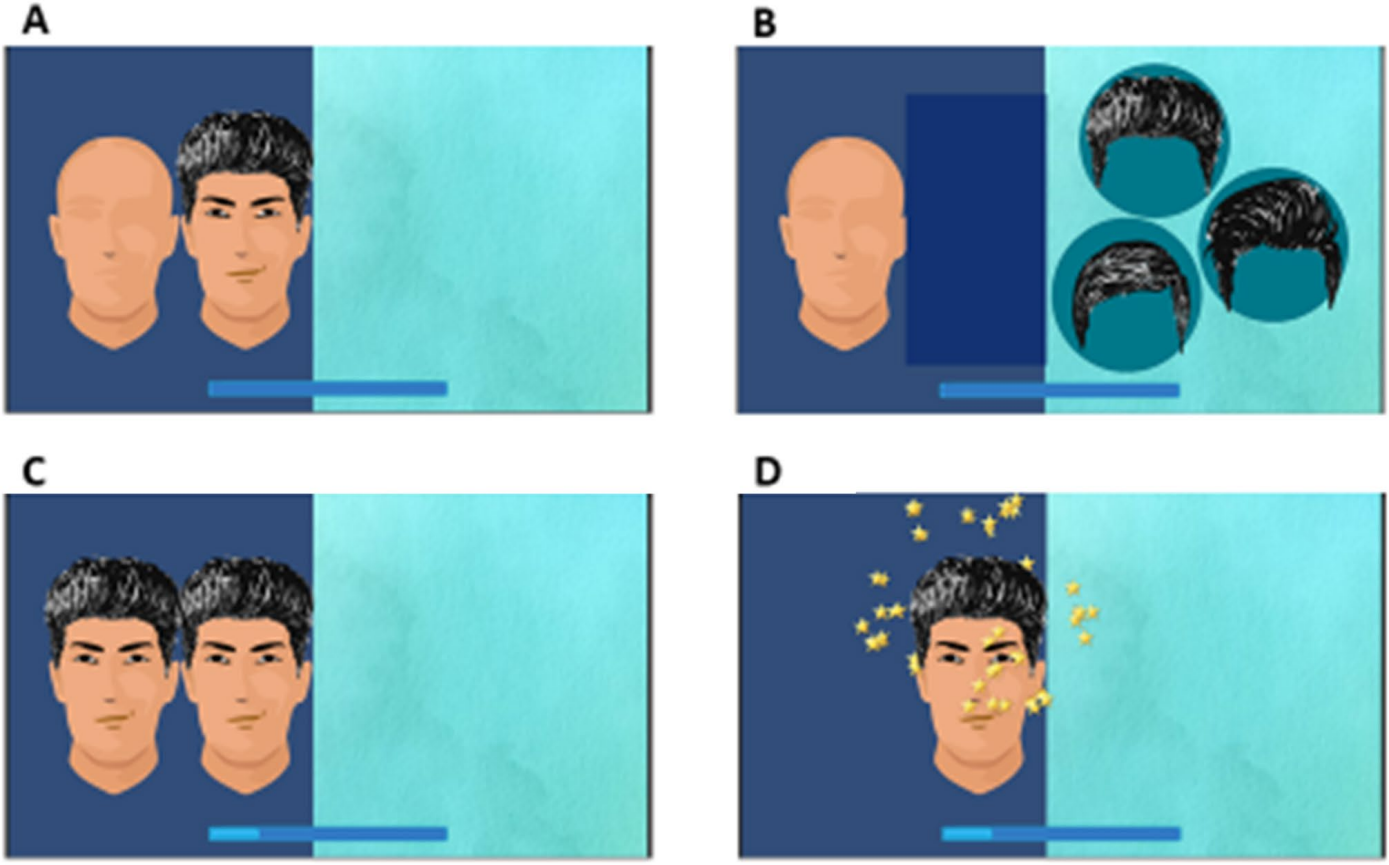
Face game gameplay. (**a**) At the start of a level, a model face and an empty face lacking facial features are shown. (**b**) After a delay the model face is covered and for each facial feature the player is shown three options, from which the player selects one to add to the empty face, in attempt to make it the same as the model face. (**c**) Once all the components are added to the empty face, the model face is revealed and the similarity between the two is assessed. (**D**) A pleasant audio-visual feedback is given if the two faces match completely.

One common symptom of ASD is impaired eye contact and ignoring facial expressions during a social interaction. We developed this game to offer practice for the identification of facial expressions along with working memory training. The objective in the game is to complete an empty face by adding facial components like hair, eyes, and mouth, such that the completed face matches a model face (Fig. 4). For easier levels, we have used less detailed, cartoonish faces. As the player progresses to more difficult levels, more details are added to the face such that the faces look more real. After trying out an initial version of the games at the center, we noticed that many children were unable to understand that they have to match the components in order to complete the empty face. To overcome this difficulty, we added an introductory level in which the player learns to match the facial components individually without any face on the screen. The introductory level was presented in three stages. Each stage had 4 sub-levels. In the first stage, the first sub-level started with a sample hair and only one option to match it. In the subsequent sub-levels, we increased the number of options. The second and the third stages included matching of eyes and mouth, respectively. All the sub-levels of stages two and three had three options from the starting of the stage. The participant had to finish all the sub-levels in one go to clear them.

#### Shape game

**Figure 5:**
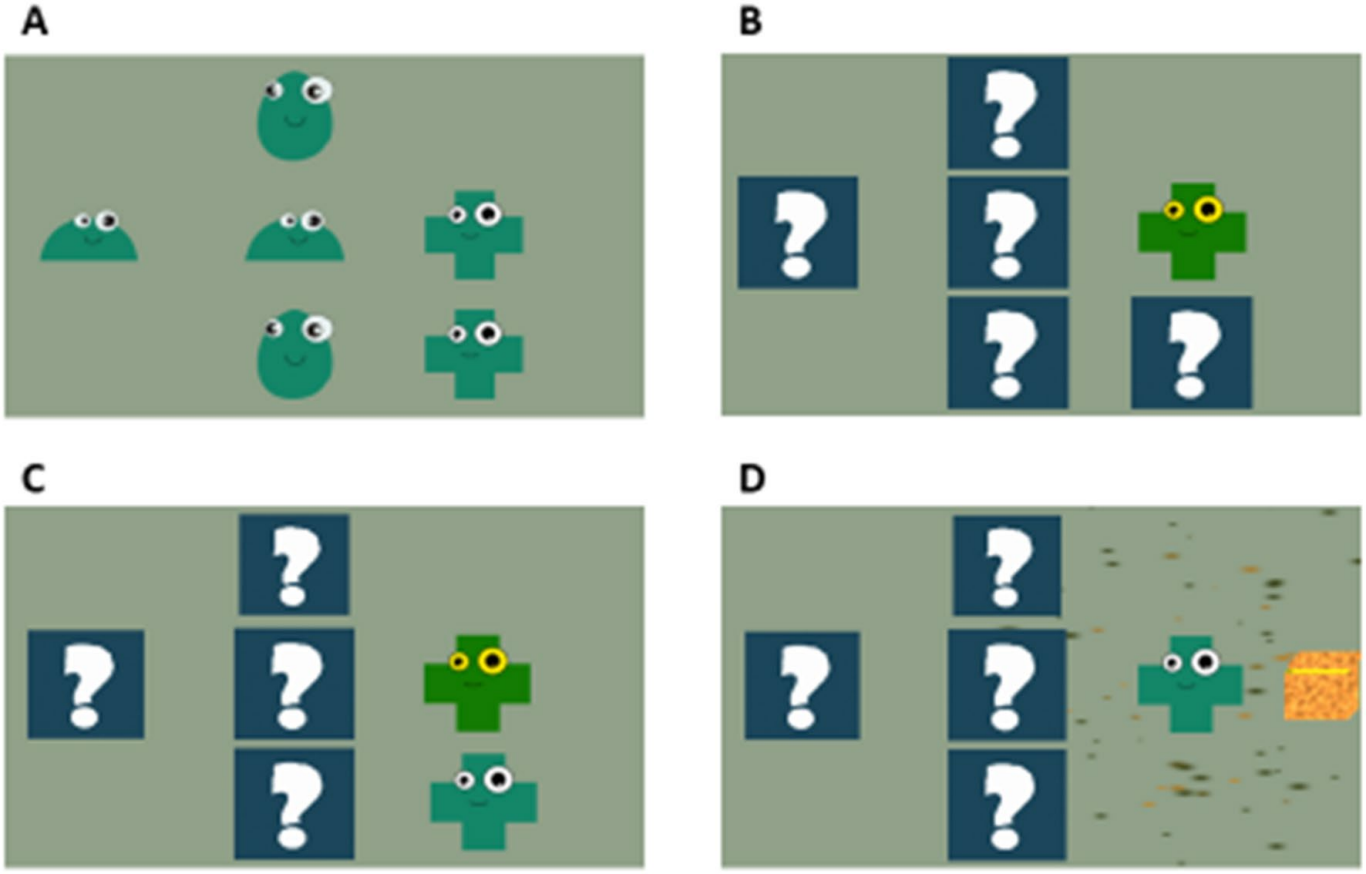
Shape game gameplay. (**A**) Objects with different shapes (two of each shape) are shown at random locations on the screen. (**B**) The player taps on one object, after which all other objects are hidden. (**C**) The player needs to find the second object with the same shape by tapping one of the hidden objects. (**D**) If the player finds the correct object, a rewarding audio-visual feedback is provided. If the selected object is wrong, all the shapes are revealed, and the play starts over again.

We designed this game especially for children who are at the lower end of the spectrum. The objective in the game is to match objects having the same shape, similar to the train game, but in this game the screen contains only simple shapes, some of which were hidden to challenge the working memory (Fig. 5). To keep the gameplay simple, no other objects were added on the screen. For making the higher levels challenging, we increase the number of objects and also shuffle their positions after a correct match.

In all the games, we have used a consistent wobbling animation to indicate which objects on the screen are supposed to be tapped when starting the game. For example, in the basket game, the bubble wobbles when it is moving in a zig-zag path to indicate that it should be tapped (the player has to use working memory to decide when to tap it).

In each game, there are multiple difficulty levels, which differed in the number of objects (visible or hidden) in the game and therefore the working memory load. If the number of errors at the current level goes beyond a certain threshold—indicating that the player is finding the game difficult—the level in the game is automatically lowered by 1 step to reduce the difficulty. On the other hand, if the player performs well at the current level, the level is increased by 1 step to increase the difficulty. This adaptive level-adjustment allows maintaining the difficulty in each game at the child’s current competence level as the child progresses through the games and prevents frustration or boredom that may be caused by very high or very low difficulty. When a child resumes the gameplay on any day, the game starts at the same level where it was left last time. The difficulty level usually increases as the player plays more, although it could occasionally decrease because of the adaptive adjustment discussed above. We recorded the maximum level reached by a player in each game during the full course of the intervention (not necessarily the last session).

### Feasibility analysis

**Figure 6:**
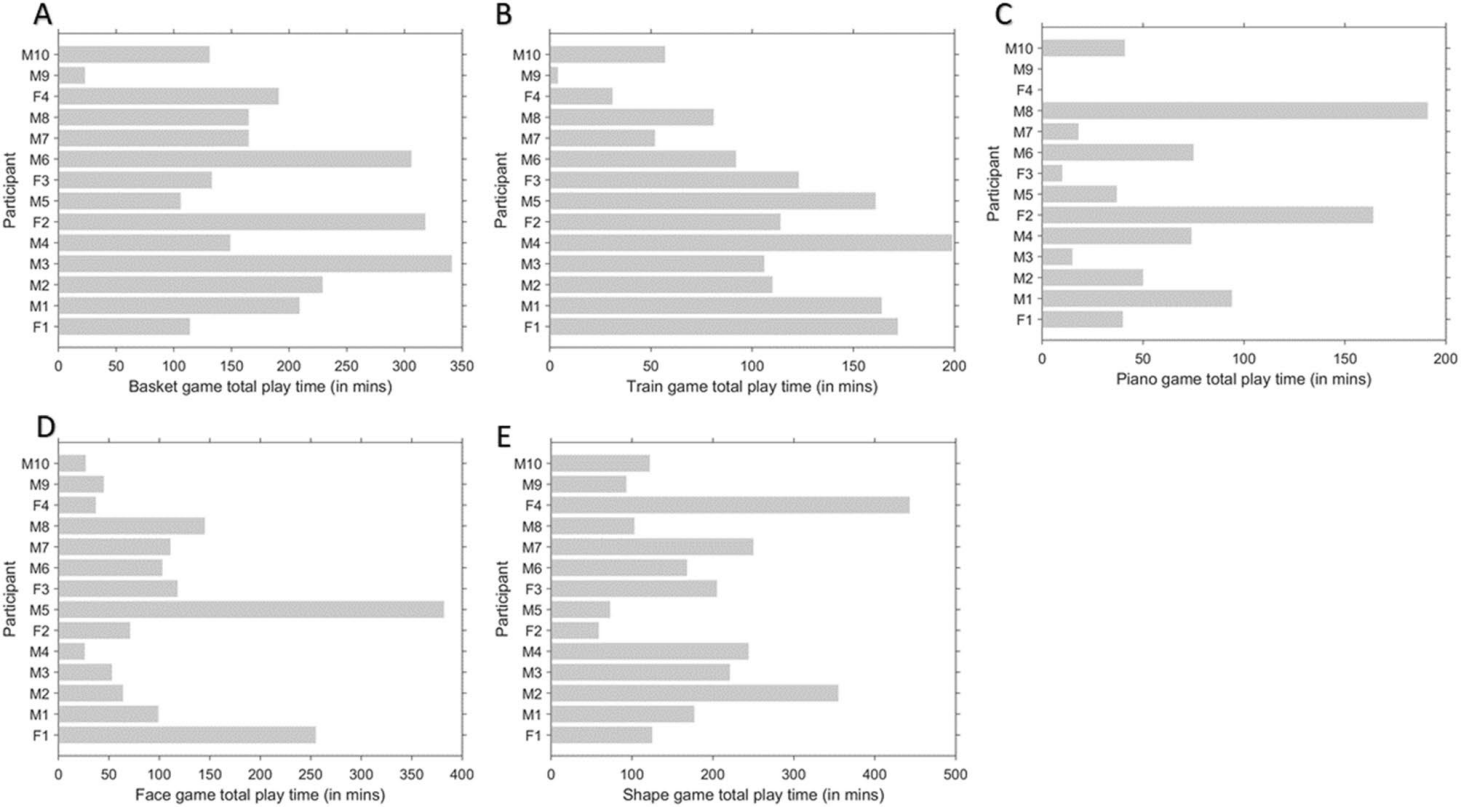
Total time spent on playing each game by the participants. Fx represents female participant numbered x and Mx represents male participant numbered x. Total time for which each participant played (in minutes) (**A**) Basket game (mean = 184.28, SD = 89.63), (**B**) Train game (mean = 104.71, SD = 56.77), (**C**) Piano game (mean = 57.78, SD = 58.41), (**D**) Face game (mean = 109.71, SD = 98.78), (**E**) Shape game (mean = 188.42, SD = 109.62).

**Figure 7:**
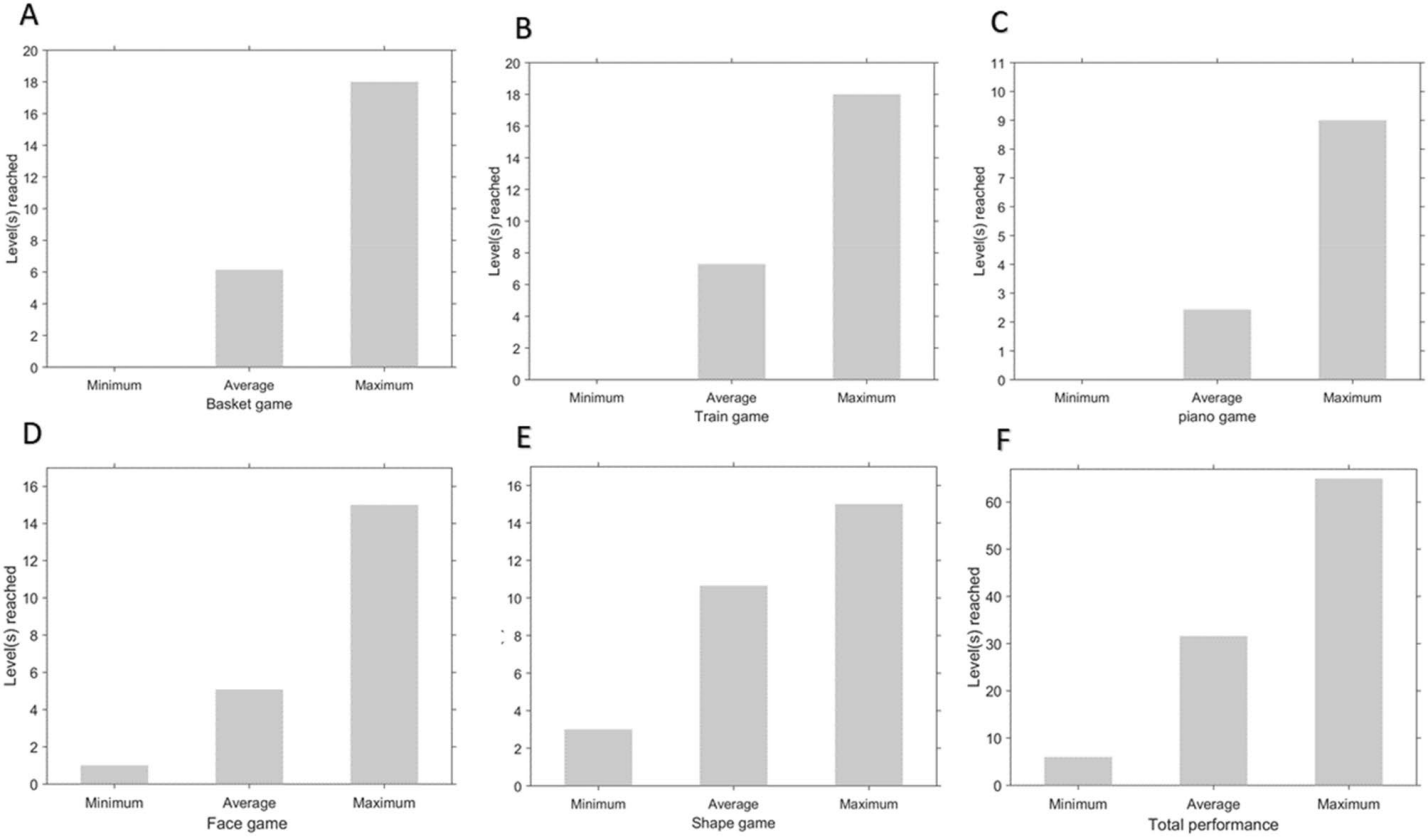
Performance in each game and total performance. The bars indicate the minimum, the average, and the maximum levels reached by the participants during the entire training duration. (**A**) Basket game (min = 0, mean = 6.14, max = 18), (**B**) Train game (min = 0, mean = 7.28, max = 18), (**C**) Piano game (min = 0, mean = 2.42, max = 9) (**D**) Face game (min = 1, mean = 5.07, max = 15), (**E**) Shape game (min = 3, mean = 10.64, max = 15), (**F**) Total performance (min = 6, mean = 31.57, max = 65). A level of 0 indicates that the participant did not start the game (Piano game) or failed to clear the introductory levels (Basket game and Train game).

During this pilot trial, we collected notes about the user-experience of the participants. We noted how the participant responded to different aspects of the games, with the aim of informing the future development of the games. These notes are summarized in Supplementary Table S2. Most of the children were able to interact with the games in the training sessions. On average, each participant had 22.78 sessions of training, and these sessions had an average duration of 28.09 min (Supplementary Figure S1). The Basket and the Shape games were the most engaging games according to the time spent by the participants in playing them. The Piano game was the least played (Fig. 6). Among the games with the introductory levels, children completed more levels in the Basket and the Train games than the Face game (Fig. 7). Among the games without the introductory levels, more levels were completed in the Shape game than the Piano game (Fig. 7).

The participants enjoyed the audio-visual feedback in the games and often reacted to it by clapping, giggling, or smiling. We found that the introductory levels in the games were typically helpful. Shape-matching was easier to understand than color-matching. Tapping was intuitive for most (except one) children. Children were able to navigate the games on their own. The notes on specific games are included in Supplementary Table S2.

### Outcome measures

**Figure 8:**
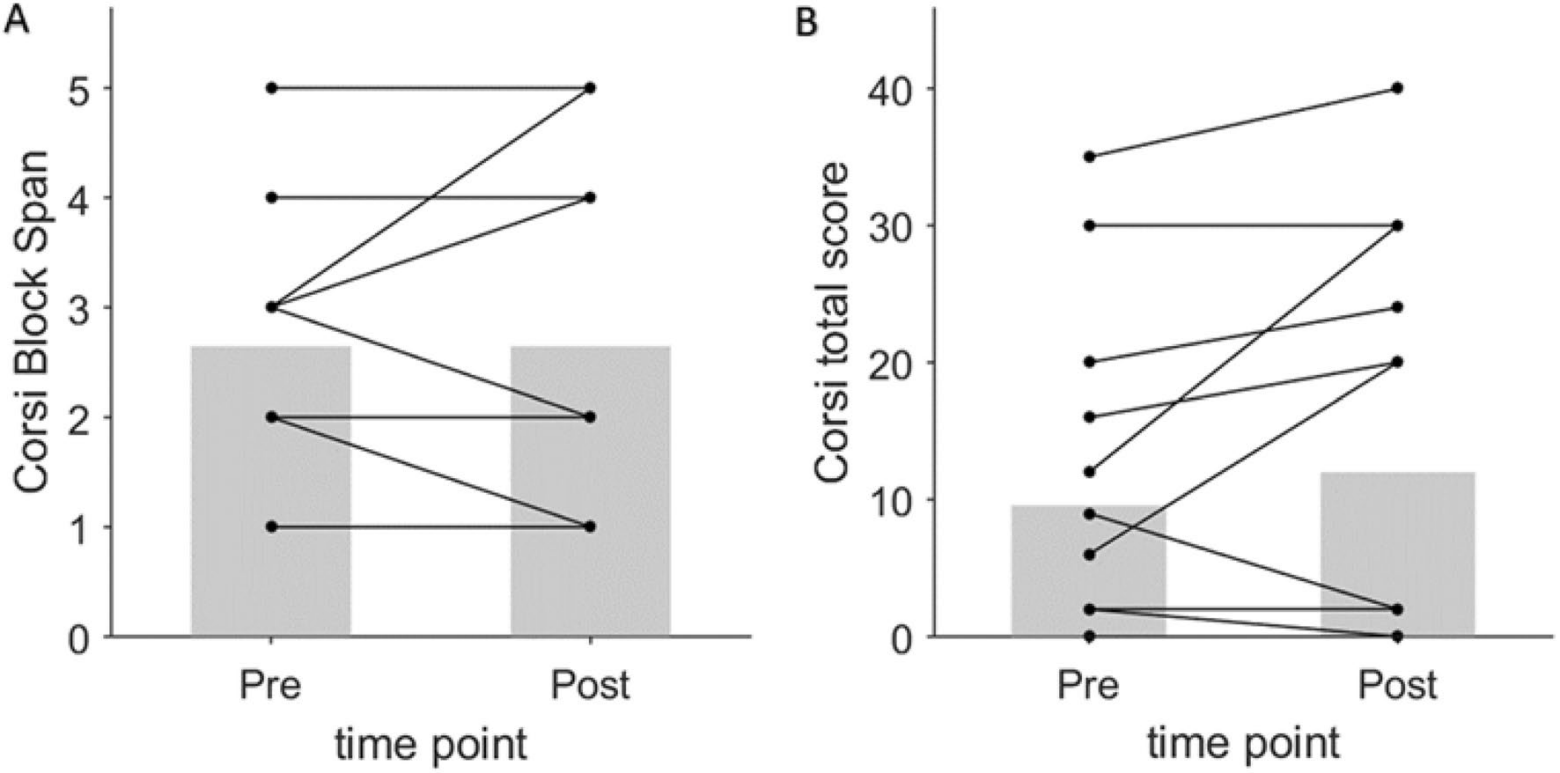
Short-term intervention with the games did not improve working memory. The graphs show pairwise comparisons between the pre-intervention and the post-intervention measures of working memory: The Corsi block span (**A**) and the Corsi total score (**B**). No significant change was observed with either measure. Both plots have N = 14 pairs of points, each corresponding to a subject, but some lines are overlapping.

**Figure 9:**
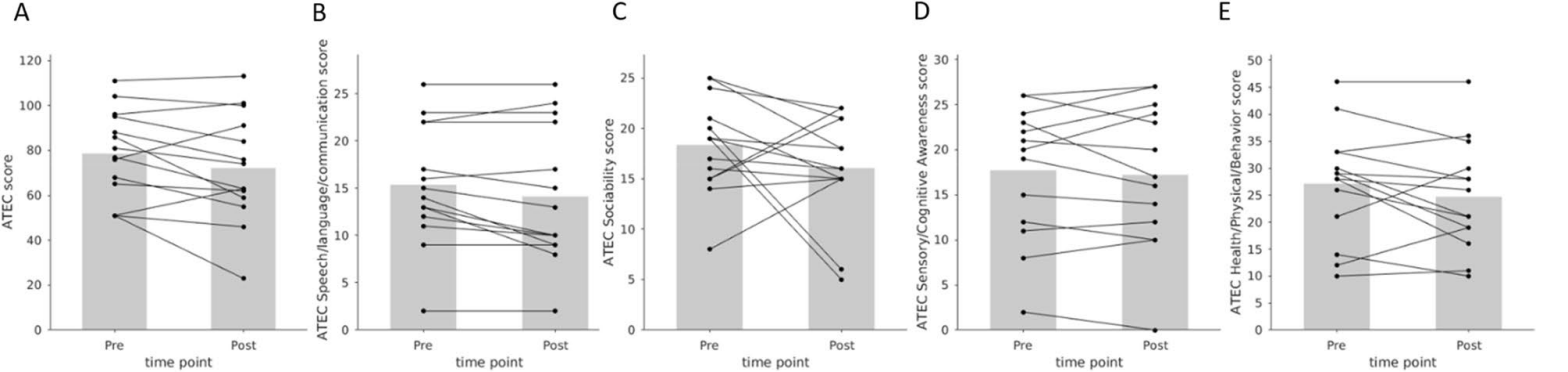
Short-term intervention with the games did not reduce autistic symptoms. The graphs show pairwise comparisons between the pre-intervention and the post-intervention measures of autistic symptoms measured using ATEC total score (**A**), or the four sub-categories of ATEC, namely Speech/Language/Communication (**B**), Sociability (**C**), Sensory/Cognitive Awareness (**D**), and Health/Physical/Behavior (**E**). No significant change was observed in any case.

The means and SDs of the 14 participating children at the pre-intervention and the post-intervention time points are presented in Table 2 and Figs. 8 and 9.

**Table 2:** Mean and standard deviation values of the outcome measures before and after the training.

Measures	Pre-intervention		Post-intervention	
	Mean	SD	Mean	SD
Corsi task: block span	2.64	1.44	2.64	1.73
Corsi task: total score	9.57	11.67	12.00	14.59
ATEC: total score	78.57	19.62	72.14	24.22
ATEC: speech/language/communication	15.35	6.35	14.14	7.22
ATEC: sociability	18.35	4.70	16.07	5.22
ATEC: sensory/cognitive awareness	17.71	7.17	17.21	7.74
ATEC: health/physical/behavior	27.14	10.25	24.71	10.04

No change (W = 7.5, n = 14, *P* = 1) was observed in the mean block spans between the pre and post intervention conditions (Fig. 8A). Similarly, no significant change was observed in the Corsi total score (W = 27, n = 14, *P* = 0.22; Fig. 8B). The ATEC score did not show a significant reduction after the intervention (W = 26, *P* = 0.10; Fig. 9A). We further checked the change in each of the four subcategories of ATEC, and found that none of them showed a significant change: Speech/Language/Communication (W = 6, *P* = 0.06, Fig. 9B); Sociability (W = 33, *P* = 0.24, Fig. 9C); Sensory/Cognitive awareness (W = 44, *P* = 0.61, Fig. 9D); Health/Physical/Behavior (W = 25, *P* = 0.16, Fig. 9E); parametric tests and effect sizes are shown in Supplementary Table S1. In summary, the children in our study did not gain significantly from the month-long game-based training in improving working memory or in reducing autistic symptoms.

Next, we checked if there was any correlation between how well a child performed on the games and his or her improvements in the outcome measures. We noticed that the performances on different games were positively correlated (Table 3); in other words, children who reached higher levels in one game also usually reached higher levels in other games. This was expected as all games were designed to test working memory using different gameplays. Therefore, instead of analyzing the performance on each game separately, we used the sum of the maximum levels in all five games achieved by a child as a measure of the performance on the games, henceforth referred to as the ‘total game performance’.

**Table 3:** Performances on the five games were correlated.

	Basket	Piano	Shape	Train
Piano	0.83 (0.00025)			
Shape	0.69 (0.0059)	0.65 (0.012)		
Train	0.86 (0.000085)	0.77 (0.0012)	0.74 (0.0027)	
Face	0.38 (0.17)	0.45 (0.10)	0.76 (0.0017)	0.55 (0.043)

Each value indicates the Spearman correlation and the corresponding *P* value (in parenthesis) between maximum levels reached in a pair of games.

**Figure 10:**
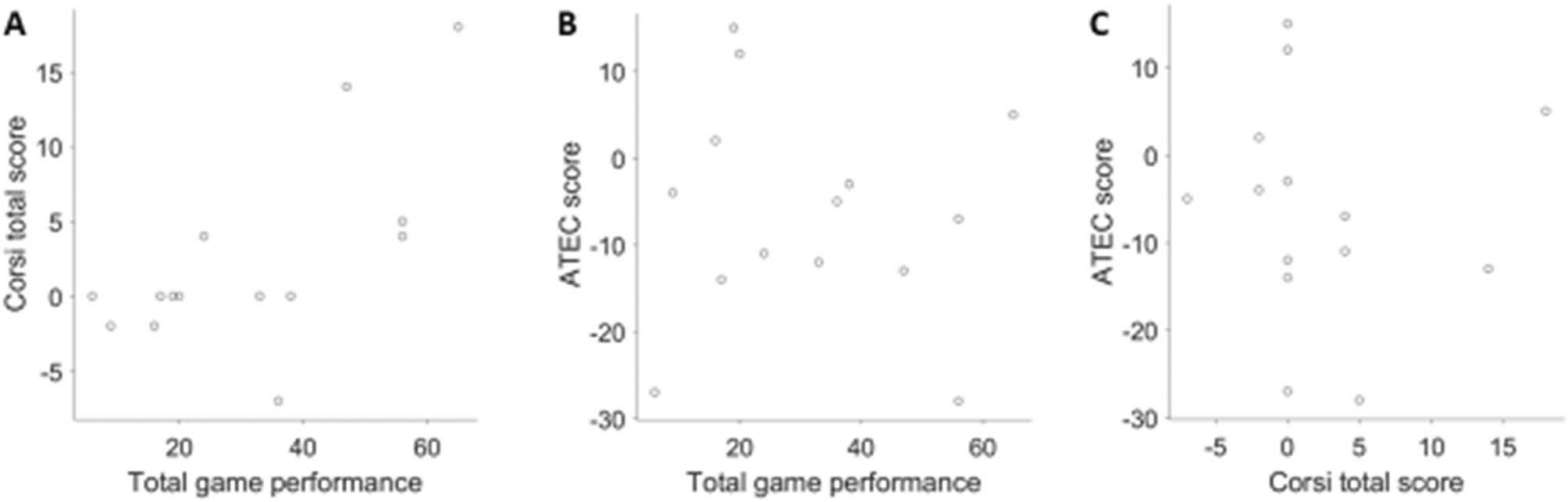
Performance on the games relates to improvements in working memory but not in autistic symptoms. (**A**) Scatter plot shows a positive correlation between the total game performance (the sum of maximum levels reached in different games) and the change in the Corsi total score with the intervention. (**B**) No correlation was seen between the total game performance and the change in the ATEC score. (**C**) Similarly, no correlation was observed between the change in the ATEC score and the change in the Corsi total score.

The total game performance showed a significant positive correlation with the change in the Corsi total score (Spearman rho = 0.68, *P* = 0.0071, n = 14; Fig. 10A). A similar positive correlation was seen between the total game performance and the change in the Corsi Block Span (Spearman rho = 0.55, *P* = 0.04, n = 14). This finding suggests that although there was no significant increase in the working memory of all children as a group after the month-long intervention, the children who performed well on the games were more likely to show an increase in their working memory. This raises the possibility that a longer training on our games might be helpful in improving the working memory.

The total game performance was not correlated with the change in the ATEC score (Spearman rho = − 0.03, *P* = 0.90; Fig. 10B). Thus, the total game performance was correlated only with the working memory improvement but not with improvement in autistic symptoms. Consistent with this observation, we also found that there was no significant correlation between the change in the ATEC score and the change in the Corsi total score (Spearman rho = − 0.25, *P* = 0.38; Fig. 10C) or the Corsi Block Span (Spearman rho = − 0.12, *P* = 0.68). Therefore, our limited month-long trial does not provide any evidence that short-term smartphone-based spatial working memory training is an effective intervention for reducing autistic symptoms. Finally, we re-analyzed our results after removing the data of one participant who was diagnosed with Down syndrome. None of the above conclusions changed by this exclusion (see Supplementary Table S3).

## Discussion

In this study, we have developed mobile-based games for training working memory in autistic children. These games are well suited for children with neurodevelopmental conditions as they are designed keeping in mind the preferences of the target population and by doing iterative user testing during the development. The games are adaptive such that the difficulty level increases when the participant performs well and decreases when the participant finds the game difficult. In most of the games, we have added a few introductory levels to help the children understand the game idea in an intuitive and engaging manner. The introductory levels contain a smaller number of elements than the main games to reduce distractions; the goals in the introductory levels are also simpler than the main games. The games are designed to require only simple interactions with the screen, such as tapping and keystrokes, which are easy enough for autistic children^50^; more complex interactions such as dragging are not used. This allows the children with problems in fine motor skills to play our games. The use of language was kept to a minimum, with only very simple English words used, so that the games can be used by children globally (in our study, children in the Hindi-speaking region of northern India were able to play the games).

We conducted a preliminary trial to evaluate the feasibility and initial efficacy of short-term (1 month) training with our games in improving the working memory and reducing autism severity. As the outcome of the trial, we measured changes in the performance of the participants on the Corsi Block Tapping task and the rating on the ATEC provided by their parents (Table 2; Supplementary Table S4). Overall, the participants did not show a significant improvement in their working memory or autistic symptoms after the short-term training. Our intervention was designed to be brief: both in the training intensity (up to 30 min per day) and the overall duration (1 month), which was relatively low as compared to the 6-week or the 1-year durations used in the previous studies^37,51^. We found that the children who performed well on our games were more likely to improve their working memory, but these gains did not extend to autistic symptoms; further, there was no correlation between the improvement in the working memory and the improvement in the autistic symptoms. As the role of working memory in ASD remains debated, the results of various interventions such as ours contribute to the resolution of the debate, and from this standpoint, negative results are as informative as positive results. It is also important to look at the results of interventions from different cultural settings. As most of the previous studies on game-based interventions in ASD have been performed in developed countries, it is not clear how their results generalize to the developing countries, such as India. The freely available, intuitive games with minimal language requirements developed in this study may help in facilitating more studies globally.

The sample size used in our trial was small (n = 14), as the trial was conducted as a pilot to estimate the effect sizes for our newly developed games before a larger trial could be planned. The previous studies of similar kinds^36,37,51^ had larger sample sizes. The Corsi Block Spans (mean of 2.6) for the study participants were relatively low at pre-intervention condition than reported in previous studies^29,51,52^ or the normative block span reported in age-matched healthy peers (Supplementary Table S5). This suggests that our participants had severe deficiency in the working memory, which combined with the short duration of the intervention might have been responsible for the lack of improvement after the training. The baseline capacity on WM has been shown to be a predictor of the progress in computerized training^53^. Hence, it would be worthwhile to study in a larger trial whether the games can be useful for children with mild to moderate deficiency in working memory. These larger studies should preferably be of longer duration to allow more time for the intervention. Another possible improvement in future trials could be to specify an optimal ordering of games during the training. For example, it may be helpful to ask the children with more severe deficits to play the easier games initially. A study by Turley-Ames et al.^54^ found that different types of training could help high-span individuals and low-span individuals. In future studies, it would be interesting to see if different games have different efficacy depending on the symptom severity of the participants. Further, in future trials using our games, it would be informative to measure other EFs (such as cognitive flexibility, planning, and inhibition) at pre- and post- intervention to investigate the possible transfer of the WM training to other EF domains. The efficacy of the games can be compared against an active control (for example, a non-WM/EF-targeting game or a non-specific app).

An emerging idea in the field, as emphasized in Green et al.^55^, is that the involvement of parent/caregiver could improve the effectiveness of the early interventions. This has been shown especially in the case of the social communication interventions. In a recent pilot trial, Diwan et al. studied the feasibility and effectiveness of a “Parent mediated intervention for Autism Spectrum Disorder Plus (PASS Plus)” treatment, where the authors conducted at-home, one-on-one therapy sessions^56^. Though they observed lower fidelity for the therapy, their method was highly feasible in rural areas with low- and middle-income country settings. Future studies on our games can involve the parent–child dyad in which the parent can conduct the training sessions in their homes. Involving parents or caregivers to conduct training session can potentially fill the treatment gap for autism.

The total time spent on playing different games (Fig. 6) shows that the Basket game and the Shape game were the most engaging for the participants. The average levels reached in different games in this pilot trial (Fig. 7) show that the Piano game and the Face game were the most difficult. In future revisions of the games, this information will be useful to update the games to improve their engagement and adjust their difficulty levels. We have also released the source-code free of any restrictions so that other researchers can further build upon the framework of these games to create new training paradigms. The current versions of the games include some design elements such as multisensory facilitation, reinforcement, and adaptive training which are proposed to be more effective at WM training^57,58^. The future versions of our games can also include other game design elements (which are missing in the current version) such as performance graphs, badges, leaderboards, and avatars, which are also shown to be motivating for the players^59^. Perceptual learning (PL) is also shown to induce brain plasticity and improve learning and attention. Feasibility and safety studies on a three-dimensional multiple object-tracking (3D-MOT) paradigm, NeuroTracker, have shown promising results for patients with traumatic brain injury^61,62^. Cognitive perceptual training using the 3D-MOT paradigm resulted in significant improvements in attention in students with neurodevelopmental conditions^63^. Intervention with gamification of PL tasks has shown broader learning in healthy individual^60^. In future revisions of our games, components of perceptual learning can be added to obtain broader gains in cognitive deficits related to ASD.

In conclusion, we developed a suite of customized games to train the working memory of children with ASD. We conducted a pilot trial to study the feasibility and initial efficacy of a short-term intervention with our games in improving the working memory and reducing the severity of autistic symptoms. In our month-long pilot study with 14 participants, we did not observe a significant improvement in their working memory or severity of autistic symptoms. However, we observed a significant positive correlation between the performance on our games and improvement in the working memory. Thus, a longer trial in which the children are able to make more progress in the games might show a more tangible improvement in working memory. Finally, we have made the games freely available on the Android Play Store. We expect them to be of help to the community of researchers and therapists working with children affected by ASD and other neurodevelopmental conditions.

## Supplementary Information

Supplementary Information.
