# Supplementary material for: Development and testing of a game-based digital intervention for working memory training in autism spectrum disorder
Source: Sci Rep. 2021 Jul 5;11:13800. doi: 10.1038/s41598-021-93258-w (PMC8257736; doi:10.1038/s41598-021-93258-w)
Supplement: Supplementary file 1 — Supplementary Information. [file 41598_2021_93258_MOESM1_ESM.pdf]

Supplementary Information

Development and testing of a game-based digital intervention for working memory training in autism spectrum disorder

Surbhit Wagle[1], Arka Ghosh[1], P Karthic[1], Akriti Ghosh[2], Tarana Pervaiz[2], Rashmi Kapoor[2,3], Koumudi Patil[4], Nitin Gupta[1,5,6,*]

1. Department of Biological Sciences and Bioengineering, Indian Institute of Technology Kanpur, Kanpur 208016, Uttar Pradesh, India

2. Amrita School for Special Children and Rehabilitation Center, Kanpur 208005, Uttar Pradesh, India

3. Regency Hospital Limited, Kanpur 208005, Uttar Pradesh, India

4. Design Program and Department of Humanities and Social Sciences, Indian Institute of Technology Kanpur, Kanpur 208016, Uttar Pradesh, India

5. Cognitive Science Program, Indian Institute of Technology Kanpur, Kanpur 208016, Uttar Pradesh, India

6. Mehta Family Center for Engineering in Medicine, Indian Institute of Technology Kanpur, Kanpur 208016, Uttar Pradesh, India

Contents:

Supplementary Figure S1

Supplementary Tables S1-S5

References

Supplementary Figure S1

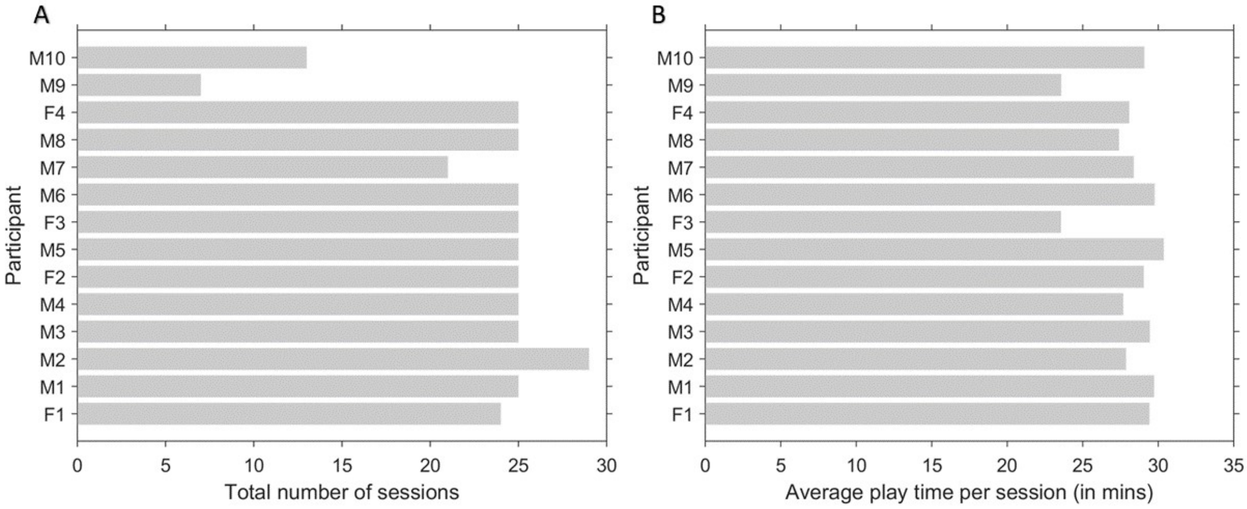

Supplementary Figure S1. Training duration for each participant. Fx represent female participant numbered x and Mx represent male participant numbered x. **(A)** Total number of training session for each participant (mean = 22.78 sessions, SD = 5.76) **(B)** Average time per session for which each participant interacted with the games (mean = 28.09 minutes, SD = 2.10).

Supplementary Table S1

	N = 14		
**Parameter**	***t***	**P-value**	**Cohen's *d***
Corsi task: Block Span	0	1	0 [a,1]
Corsi task: Total score	1.39	0.18	0.37 [a,3]
ATEC: Total score	-1.91	0.07	0.51[b,4]
ATEC: Speech/Language/Communication	-2.18	0.04*	0.58 [b,4]
ATEC: Sociability	-1.29	0.21	0.34 [b,3]
ATEC: Sensory/Cognitive Awareness	-0.64	0.53	0.17 [b,2]
ATEC: Health/Physical/Behavior	-1.48	0.16	0.39 [b,3]

Supplementary Table S1: The results from the parametric *t*-test. *P-value <0.05. a the original value is reported (post mean >= pre mean). b the absolute value is reported, original values are negative (post mean < = pre mean). 1 zero effect size, Cohen's (1988)[1] convention for zero effect size (d=0), no difference in mean. 2 very small effect size, Cohen's (1988)[1] convention for very small size (d < 0.2). 3 small effect size, Cohen's (1988)[1] convention for small size (d = 0.2). 4 medium effect size, Cohen's(1988)[1] convention for medium size (d = 0.5)

Supplementary Table S2

Observation	Audiovisual positive reinforcement	Understanding the game objective	Interaction with the games	User-interface and graphic design
General Feedback	All the participants enjoyed the audiovisual feedback. They showed their happiness by clapping, giggling or smiling.	The introductory levels helped most of the participants in understanding the game objective. In general, shape-matching was easier to understand than color-matching	Most of the participants were able to tap. Only one participant often tried to drag objects to match them. The wobbling effect helped the participants in identifying objects that can be tapped.	The participants found the game elements and the interface easy to navigate. They were able to change game on their own and start a level.
Specific Feedback	* Piano game: Participants did not respond much to the tones played as part of the game objective	* Basket game: 3 out of 14 participants did not finish the introductory level.<br>* Train game: 13 out of 14 understood the game objective. However, 3 of these 13 participants did not make progress in the game, possibly because the extra elements/objects in the main level were distracting for them.<br>* Face game: 13 out of 14 cleared the introductory levels and made progress in the game.<br>* Piano game, Shape game: Participants progressed in the game without any introductory levels.	* Basket game: 4 out of 14 participants initially tried to drag the fruit into the basket. Later 3 of them switched to tap and 1 did not. For 2 out of 14 participants, the movement of the fruit was very fast which led to mistakes in the game.<br>* Piano game: One participant tried to correct a mistake they made during the game but the games did not support any immediate correction.	* Piano game: One participant got fixated on the red-color piano tile and tapped only the red-colored tile.

Supplementary Table S2: Observations made by the experimenters during the training sessions.

Supplementary Table S3

	With all 14 participants		With 13 participants with ASD	
Parameter	Signed Rank	P-value	Signed Rank	P-value
Corsi task: Block Span	7.5	1	7.5	1
Corsi task: Total score	27	0.22	20	0.32
ATEC: Total score	26	0.10	26	0.18
ATEC: Speech/Language/Communication	6	0.06	6	0.06
ATEC: Sociability	33	0.24	33	0.40
ATEC: Sensory/Cognitive Awareness	44	0.61	40.5	0.75
ATEC: Health/Physical/Behavior	25	0.16	25	0.29
Parameter	Spearman rho	P-value	Spearman rho	P-value
total game performance vs change Corsi total score	0.68	0.007*	0.67	0.01*
total game performance vs change Corsi block span	0.55	0.04*	0.65	0.015*
total game performance vs ATEC change	-0.03	0.9	0.1331	0.66
change ATEC vs change Corsi total score	-0.25	0.38	0.014	0.96
change ATEC vs change Corsi Block Span	-0.12	0.68	0.035	0.90

Supplementary Table S3: The comparison of signed-rank tests and spearman correlations of the participants before and after removing the single participant with Down syndrome. None of the conclusions changed significantly after removing the participant. *P-value <0.05

Supplementary Table S4

ATEC distribution		
ASD severity	Pre	Post
Mild (20-49)	0	2
Moderate (50-79)	7	7
Severe (>=80)	7	5

Supplementary Table S4: Distribution of the autistic symptom severity of the study participants at the pre-intervention and the post-intervention timepoints based on the classification used in Mahapatra et al. 2020[2].

Supplementary Table S5

Corsi Block Tapping Task performance comparison to normative data			
	Corsi Block Span		
Age	Pre<br>Mean (SD)	Post<br>Mean (SD)	Normative data<br>Mean (SD)
6	1.0 (0.0)	1.0 (0.0)	N.A.
7	1.0 (0.0)	1.0 (0.0)	5.0 (0.8)
8	4.0 (0.0)	4.0 (0.0)	5.2 (1.0)
9	2.0 (0.0)	1.0 (0.0)	5.5 (1.0)
10	2.0 (1.0)	2.0 (1.73)	6.0 (1.1)
11	4.0 (0.0)	4.0 (0.0)	6.3 (0.9)
12	2.8 (1.48)	3.0 (1.87)	6.3 (0.9)
13	5.0 (0.0)	5.0 (0.0)	6.4 (0.9)

Supplementary Table S5: Comparison of the performance on the Corsi task by the study participants with normative data reported in Pagulayan et al. 2006[3]. N.A. data not available in the reference study.

References

1. Cohen, J. *Statistical Power Analysis for the Behavioral Sciences*. *Statistical Power Analysis for the Behavioral Sciences* (Lawrence Erlbaum Associates, Publishers, 1988). doi:10.4324/9780203771587.

2. Mahapatra, S. *et al.* Longitudinal Epidemiological Study of Autism Subgroups Using Autism Treatment Evaluation Checklist (ATEC) Score. *J. Autism Dev. Disord.* **50**, 1497–1508 (2020).

3. Farrell Pagulayan, K., Busch, R., Medina, K., Bartok, J. & Krikorian, R. Developmental normative data for the Corsi Block-Tapping task. *J. Clin. Exp. Neuropsychol.* **28**, 1043–1052 (2006).
